# A C≡C Triple Bond as a Structural Anchor of Planar Pentacoordinate Carbon

**DOI:** 10.1021/acs.jpca.5c07530

**Published:** 2026-03-03

**Authors:** Chagan Dari, Guang-ren Na, Li-juan Cui, Fernando Martínez-Villarino, Luz Diego, Jorge Barroso, Gabriel Merino, Zhong-hua Cui

**Affiliations:** † Institute of Atomic and Molecular Physics, 12510 Jilin University, Changchun 130023, China; ‡ Departamento de Física Aplicada, Centro de Investigación y de Estudios Avanzados Unidad Mérida, Km 6 Antigua Carretera a Progreso. Apdo. Postal 73, Cordemex, Mérida 97310, Mexico; § Doctorado en Fisicoquímica Molecular, Facultad de Ciencias Exactas, 28087 Universidad Andres Bello, Av. República 275, Santiago 8370146, Chile; ∥ Department of Chemistry, Clemson University, Clemson, South Carolina 29634, United States; ⊥ State Key Laboratory of Inorganic Synthesis and Preparative Chemistry, Jilin University, Changchun 130023, China

## Abstract

The potential energy surface of C_2_Li_4_H_3_^–^ was examined to identify stable structures containing planar hypercoordinate carbon centers. The lowest-energy form corresponds to a *C*_2*v*_-symmetric arrangement in which a Li_4_H_3_ framework encloses a C≡C triple bond. Bonding analyses (NBO, AdNDP, EDA, and IQA) show that the system consists of a C_2_^2–^ fragment interacting mainly through electrostatic attraction and weak covalent contributions with the Li_4_H_3_^+^ unit. Magnetic response calculations indicate localized electron density supported by the C≡C bond, contrasting with the delocalization mechanisms typically invoked for planar hypercoordinate carbons. Born–Oppenheimer molecular dynamics simulations confirm structural stability under thermal conditions. These results outline an alternative electronic route to achieve planar pentacoordination in carbon systems.

## Graphical abstract

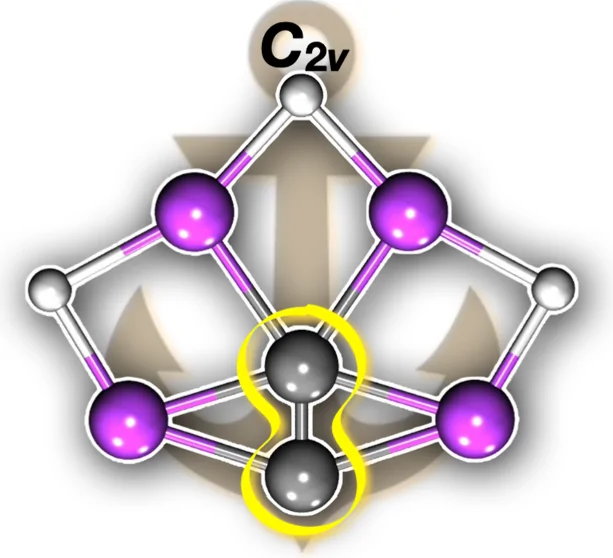

## Introduction

The concept of planar tetracoordinate carbon (ptC) was first proposed by Monkhorst in 1968 as a transition state in the stereomutation of methane.^1^ Initially regarded as a theoretical oddity, the seminal work of Hoffmann, Alder, and Wilcox foundedthe electronic criteria necessary for stabilizing such structures.^2^ Their analysis, while formally contradicting the tetrahedral paradigm established by van’t Hoff and Le Bel,^3,4^ effectively revisited the pre-stereochemical notion of planar carbon and extended it to the theoretical limits of bonding, thereby redefining the conditions under which such “aberrant” geometries could exist. A decisive step forward came with Schleyer and co-workers’ prediction of 1,1-dilithiocyclopropane,^5^ showing that ptC atoms can correspond to a true minimum on the potential energy surface (PES). Since then, numerous ptC species have been theoretically designed and, in some cases, experimentally detected or synthesized.^6–9^

These concepts were subsequently extended to higher coordination numbers, leading to planar penta- (ppC) and hexacoordinate carbon (phC) centers, as well as analogous motifs involving other main-group elements.^7,10,11^ One of the most effective stabilization strategies involves delocalization of the nonbonding *p*_π_ electrons of the central atom through π acceptor/σ donor ligands. This mechanism is most effective when the total electron count approaches 18, providing an optimal balance for multicenter delocalization and geometric symmetry, although stable species with higher or lower electron counts are also known.^5^ Representative examples include the C©Al_4_^2–^ (*D*_4*h*_) as ptC,^12^ C©Al_5_^+^ (*D*_5*h*_),^10^ and counterion-stabilized C©Be_5_^4–^ species (*D*_5*h*_) as ppCs,^13–16^ together defining a broad family of planar hypercoordinate carbon systems.^8,17–21^

An alternative stabilization strategy relies on localizing multiple bonds between the central carbon and selected ligands. This approach enhances directional bonding by reinforcing electron localization, although it often reduces molecular symmetry.^22–24^ Our group and others have successfully applied this concept to diverse systems, including ptCs such as C©BAl_3_^2–^,^22^ C©EAl_3_ (E = N–Bi),^25^ C©Li_3_E (E = As, Sb, Bi),^26^ and C_2_Al_4_,^27^ as well as phCs in C©E_3_M_3_^+^ clusters (E = S, Se, Te; M = Li–Cs).^11^ These species frequently exhibit C–E and C–C double bonds, highlighting the adaptability of carbon in nonclassical environments.^28,29^

Based on these precedents, we asked whether planar hypercoordination could be extended to systems incorporating a C≡C triple bond. In this work, we explore the PES of C_2_Li_4_H_3_^–^. The putative global minimum corresponds to a *C*_2*v*_ structure in which a C≡C unit is embedded within a Li_4_H_3_ framework containing bridging hydrogen atoms. This structure represents, to our knowledge, the first ppC system featuring a C≡C moiety. Further analyses indicate that the C_2_^2–^ fragment interacts primarily through electrostatic forces and weak covalent interactions with the Li_4_H_3_^+^ fragment. These results broaden the conceptual boundaries of planar hypercoordination and show the potential of incorporating unsaturated carbon fragments into planar architectures.

## Computational Details

The PES of C_2_Li_4_H_3_^–^ was explored for both singlet and triplet spin states using the *GASA* program (Genetic Algorithm and Structure-driven Approaches), an in-house global-optimization code for atomic clusters that combines genetic algorithms with structure-driven search strategies.^30^ Low-lying isomers within 50 kcal/mol of the global minimum were reoptimized at the PBE0-D3/aug-cc-pVTZ^31–33^ level and vibrational frequency analyses were carried out to confirm the nature of the stationary points. Single-point energy refinements were then performed using the CCSD­(T)^34^/aug-cc-pVTZ level. All calculations were performed with *Gaussian 16*.^35^ For the global-minimum ppC structure, natural bonding orbital (NBO)^36^ and adaptive natural density partitioning (AdNDP)^37^ analyses were conducted at the PBE0-D3/aug-cc-pVTZ level, with the latter performed using the *Multiwfn* program.^38^ Dynamic stability of the global minimum was assessed through Born–Oppenheimer molecular dynamics (BOMD)^39^ simulations.

To further characterize the bonding, energy decomposition analysis (EDA) à la Morokuma–Ziegler–Rauk^40^ combined with natural orbital for chemical valence (NOCV)^41^ theory was carried out using the *ADF 2020* package.^42^

Electron delocalization was examined through the induced magnetic field (**B** ^ind^)^43–45^ and the induced current-density (**J** ^ind^)^46–48^ computed with the Aromagnetic^49^ and GIMIC programs,^46,48^ respectively. These properties were evaluated on a rectangular grid centered on the molecule under a magnetic field applied perpendicular to the molecular plane. The BHandHLYP^50^/def2-TZVP level was used for these magnetic response calculations.

Finally, to obtain a detailed description of the pentacoordination at the central carbon atom, interacting quantum atoms (IQA)^51–54^ analysis was performed at the PBE0/def2-TZVP//MP2 ^55^/def2-TZVP level using the *AIMAll* program.^56^ The IQA framework decomposes the interatomic interaction energy, *V* ^IQA^, into Coulombic, *V*_C_, and exchange-correlation, *V*_XC_, components, where *V*_C_ represents primarily electrostatic-type contributions and *V*_XC_ reflects covalent-type interactions.

## Results and Discussion

Figure 1 shows the low-lying structures of C_2_Li_4_H_3_^–^ computed at the CCSD­(T)/aug-cc-pVTZ//PBE0-D3/aug-cc-pVTZ level. The putative global minimum is a singlet with *C*_2*v*_ symmetry, featuring a ppC at the center of a Li_4_H_3_ framework. As detailed below, the ppC motif is predominantly stabilized by electrostatic interactions along with only weak covalent contributions. This ppC structure lies 5.2 kcal/mol below the next most stable isomer, a ptC species.

**Figure 1:**
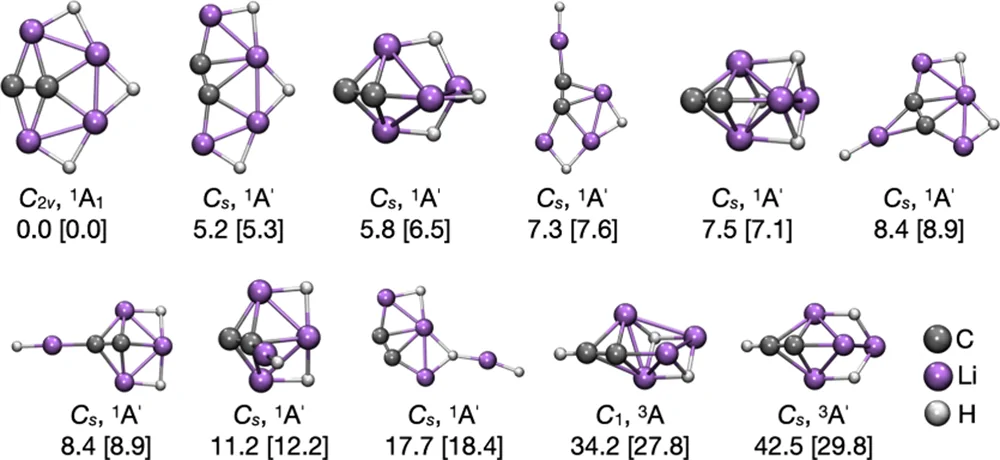
Low-lying energy isomers of C_2_Li_4_H_3_^–^. Relative energies (kcal/mol) including zero-point energy (ZPE) corrections computed at the CCSD­(T)/aug-cc-pVTZ//PBE0-D3/aug-cc-pVTZ level and at the PBE0-D3/aug-cc-pVTZ level (given in square brackets).

In addition, other low-lying singlet isomers retain the C_2_ fragment and adopt *C*_*s*_ symmetry, forming either quasi-planar or three-dimensional arrangements. The lowest triplet structure is nonplanar and lies 34.2 kcal/mol above the global minimum. The *T*_1_ diagnostics^57^ for all CCSD wave functions fall within acceptable reference thresholds (0.013–0.015 for singlets; 0.047–0.056 for triplets), confirming the reliability of single-reference methods for these species. Reoptimization of the global minimum at the CCSD­(T)/aug-cc-pVTZ level yielded a lowest vibrational frequency of 9 cm^–1^, supporting the accuracy of the chosen PBE0-D3/aug-cc-pVTZ level (Figure S1).

The C–C bond length (1.254 Å) agrees with a typical C≡C triple bond (approximately 1.26 Å), and the Wiberg bond index^58^ (WBI = 2.97) confirms its triple-bond character. The C–Li distances (2.077 and 2.164 Å) correspond to single bonds according to the Pyykkö-Atsumi covalent radii,^59^ while their low WBI values (0.06–0.07) indicate predominantly electrostatic character. The Li–H distances (1.747–1.883 Å) with WBIs near 0.16 suggest weak, partially electrostatic interactions.

The AdNDP^37^ analysis identifies three 2c–2e bonds between the carbon atoms, one σ and two π, with occupation numbers (ONs) close to 2.00 |e|, confirming a C≡C triple bond (Figure 2). The bent Li_4_H_3_ framework is stabilized by three 3c–2e Li–H–Li bonds (ONs = 1.97–1.99 |e|). The remaining four electrons can be represented either as two weakly delocalized 4c–2e σ bonds (ONs = 1.99–2.00 |e|) or, alternatively, as two localized 1c–2e lone pairs with ONs from 1.86 to 1.88 |e| (Figure S2). The reduced occupation numbers in the localized description indicate partial electron delocalization toward the Li_4_H_3_ fragment. We therefore adopt the 4c–2e representation, which better reflects the actual extent of electron sharing between the C_2_ unit and the surrounding framework.

**Figure 2:**
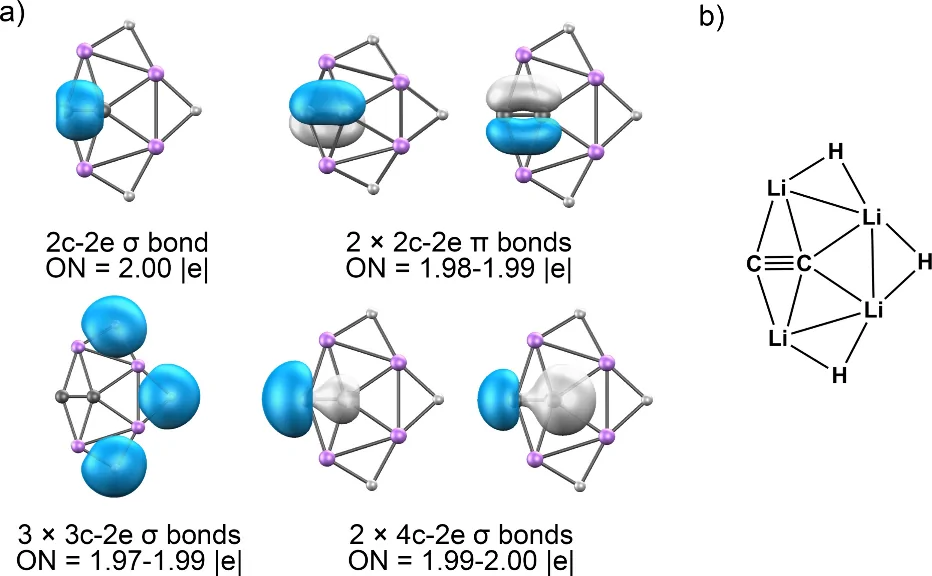
(a) AdNDP analysis of C_2_Li_4_H_3_^–^ at the PBE0-D3/aug-cc-pVTZ level. Occupation numbers (ON) are given in |e|. (b) Schematic representation of C_2_Li_4_H_3_^–^.

To verify that the central carbon is indeed pentacoordinate, we examined the bonding interactions using EDA à la Morokuma–Ziegler–Rauk and the interacting quantum atoms (IQA) method. In the EDA framework, the optimal fragmentation scheme (singlet C_2_^2–^ + singlet Li_4_H_3_^+^, see Table S1) yielded the lowest orbital interaction energy (Δ*E*_oi_) and Pauli-repulsion, Δ*E*_Pauli_ terms. The EDA results show that the electrostatic component, Δ*E*_elstat_, accounts for 84.8% of the total attractive interaction, while the orbital contribution, Δ*E*_oi_, represents 15.1% (Table 1), confirming a predominantly electrostatic interaction between C_2_^2–^ and Li_4_H_3_^+^.

**Table 1:** Energy Decomposition Analysis (EDA) of C_2_Li_4_H_3_^–^ Using C_2_^2–^ (Singlet) and Li_4_H_3_^+^ (Singlet) Fragments, Computed at the PBE0-D3/TZ2P-ZORA Level^a^

contribution	interaction	C_2_^2–^ + Li_4_H_3_^+^
Δ*E*_int_		–408.0
Δ*E*_Pauli_		123.7
Δ*E*_disp_		–0.6 (0.1%)
Δ*E*_elstat_ ^b^		–450.8 (84.8%)
Δ*E*_oi_ ^b^		–80.4 (15.1%)
Δ*E*_oi(1)_ ^c^	C_2_^2–^ → Li_4_H_3_^+^ σ donation	–33.0 (41.0%)
Δ*E*_oi(2)_ ^c^	C_2_^2–^ → Li_4_H_3_^+^ π donation	–11.8 (14.7%)
Δ*E*_oi(rest)_ ^c^		–35.6 (44.3%)

a Energies are reported in kcal/mol.

b The percentage contribution with respect to total attraction is given in parentheses.

c The percentage contribution in parentheses is given with respect to total orbital interaction.

The natural population analysis (NPA)^60^ shows a charge of approximately −1.0 |e| on the ppC center and −0.7 |e| on the adjacent carbon, while each surrounding Li atom carries a charge of about +0.8 |e| and each hydrogen atom about −0.8 |e|. These results indicate that electrostatic interactions, rather than extensive delocalization, dominate the stabilization of the ppC framework, confirming that the selected EDA fragmentation is appropriate.

A similar picture was previously reported for C_6_Li_6_, where the global minimum was described as three C_2_^2–^ fragments interconnected through lithium bridges with predominantly electrostatic C–Li interactions.^61^ This resemblance emphasizes the role of C_2_^2–^ units as fundamental building blocks in lithium-stabilized carbon clusters.

Further decomposition of Δ*E*_oi_ using the EDA-NOCV scheme reveals three significant orbital interactions, visualized through deformation densities (Δρ) depicting electron flow from donor to acceptor orbitals (red to blue). As shown in Figure S3, two dominant contributions, accounting for approximately 56% of the total Δ*E*_oi_, correspond to σ- and in-plane π-donations from localized orbitals of C_2_^2–^ into vacant σ-orbitals of Li_4_H_3_^+^, confirming a largely electrostatic but weakly covalent character for the C_2_–Li_4_H_3_ interaction.

The IQA further clarifies the bonding nature by partitioning the interatomic interaction energy into Coulombic, exchange, and correlation terms (Table 2 and Figure 3). The C–C interaction is strongly attractive due to the exchange contribution (−450.6 kcal/mol), consistent with covalent bonding, whereas the C–Li interactions are dominated by the Coulomb term (−139.7 and −159.9 kcal/mol) with small exchange components (−9.6 and −10.7 kcal/mol), reflecting their primarily electrostatic character. This residual exchange–correlation stabilization arises mainly from σ- and in-plane π-donations from the C_2_^2–^ fragment into vacant σ-type acceptor orbitals of the Li_4_H_3_^+^ framework (Figure S3).

**Figure 3:**
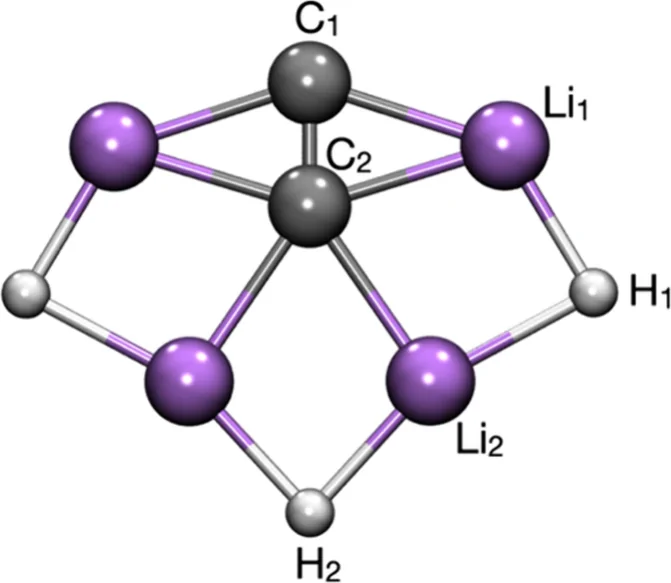
Global minimum structure of C_2_Li_4_H_3_^–^ at the CCSD­(T)/aug-cc-pVTZ//PBE0-D3/aug-cc-pVTZ level, showing the atom numbering used throughout the analysis.

**Table 2:** Energy Components (kcal/mol) from the IQA Analysis of C_2_Li_4_H_3_^–^ ^a^

	C_2_–C_1_	C_2_–Li_1_	C_2_–Li_2_	C_1_–Li_1_	Li_1_–H_1_	H_1_–Li_2_	Li_2_–H_2_	Li_1_–Li_2_	Li_2_–Li_2_
Δ*E*_IQA_	0.0								
*V*_IQA_^Int^	–320.0	–149.3	–170.6	–100.4	–153.9	–139.4	–146.9	101.2	102.5
*V*_C_^Int^	130.6	–139.7	–159.9	–90.7	–138.9	–128.5	–134.1	101.5	102.9
*V*_XC_^Int^	–450.6	–9.6	–10.7	–9.7	–15.0	–10.9	–12.9	–0.3	–0.4

a *V*_IQA_^int^, *V*_C_^int^, and *V*_XC_^int^ are the interatomic interaction energies and their Coulombic, exchange, and correlation energy components, respectively. Δ*E*_IQA_ denotes the total integration error in the IQA energies.

To evaluate whether the bonding in C_2_Li_4_H_3_^–^ follows a localized scheme, we examined its magnetic response under an external magnetic field applied perpendicular to the molecular plane. The induced current density (**J** ^ind^) reveals localized currents around the Li and C nuclei (Figure 4a), indicating the absence of continuous ring currents typically obtained in delocalized π systems. This finding is consistent with the induced magnetic field (**B** ^ind^) analysis, whose *z*-component (*B* ^ind^_*z*_) exhibits localized shielding cones. The only cone involving more than one atom corresponds to the C_2_ fragment, as expected for the delocalized π electrons of the C≡C bond (Figure 4b).

**Figure 4:**
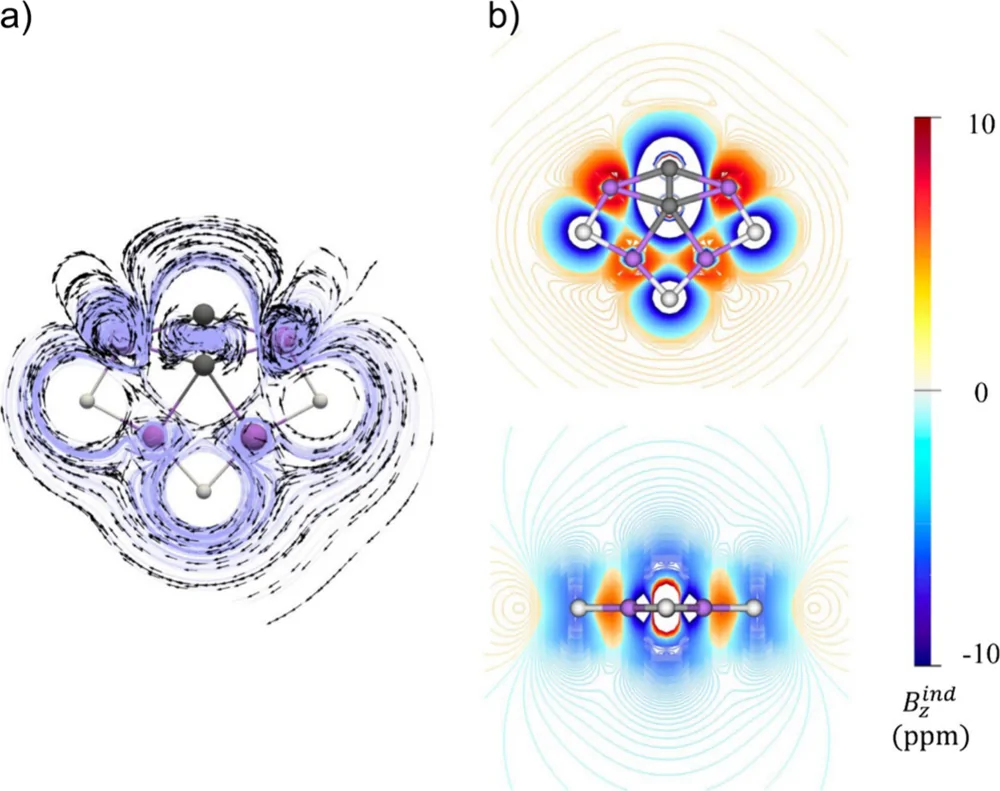
(a) Induced current-density (**J** ^ind^) vector maps and (b) isolines of the *z*-component of the induced magnetic field (*B* ^ind^_*z*_) for the global minimum of C_2_Li_4_H_3_^–^, computed on the molecular plane.

The thermal stability of the ppC structure was assessed using Born–Oppenheimer molecular dynamics (BOMD)^40^ simulations with a 0.5 fs time step for 10 ps trajectories at 300 and 400 K. The molecule preserved its planarity and connectivity (Figure S4), without any sign of isomerization or distortion. Root-mean-square deviation (RMSD) analysis revealed minimal atomic motion, confirming the robustness of the ppC framework.

Finally, the photoelectron spectrum of C_2_Li_4_H_3_^–^ was simulated using the P3+ approach to facilitate future experimental identification.^62,63^ The vertical detachment energy (VDE) of the ground-state transition is 3.58 eV, while the first excited state appears at 4.15 eV, giving a spectral gap of 0.57 eV. These features provide distinctive signatures for potential gas-phase detection via photoelectron spectroscopy.

## Conclusions

In this work, we characterized the global minimum of C_2_Li_4_H_3_^–^, a species with *C*_2*v*_ symmetry featuring a bent Li_4_H_3_ framework surrounding a C≡C triple bond. This structure contains a planar pentacoordinate carbon (ppC) as the central atom, an unprecedented arrangement in hypercoordinate carbon chemistry. Magnetic response analyses reveal strong electron localization, in sharp contrast to the delocalization mechanisms commonly invoked to stabilize planar hypercoordinate centers. Bonding analyses based on NBO, AdNDP, EDA, and IQA consistently confirm the presence of a triple-bonded C_2_^2–^ fragment interacting predominantly through electrostatic forces and weak covalent contributions with the Li_4_H_3_^+^ fragment. Born–Oppenheimer dynamics simulations further show that this ppC structure is dynamically stable under thermal conditions. Together, these results establish a new bonding motif in which a C≡C triple bond enables planar pentacoordination, extending the boundaries of carbon bonding and redefining the electronic scope of hypercoordinate frameworks.

## Supplementary Material



## Notes

Optimized structures of C_2_Li_4_H_3_^–^, BOMD simulations, and Cartesian coordinates (PDF)
