# Supplementary material for: A C≡C Triple Bond as a Structural Anchor of Planar Pentacoordinate Carbon
Source: J Phys Chem A. 2026 Mar 3;130(10):2091–6. doi: 10.1021/acs.jpca.5c07530 (PMC13730438; doi:10.1021/acs.jpca.5c07530)
Supplement: Supplementary file 1 [file jp5c07530_si_001.pdf]

Supporting Information for

A C≡C Triple Bond as a Structural Anchor of Planar Pentacoordinate Carbon

Chagan Dari,[a] Guang-ren Na,[a] Li-juan Cui,[a] Fernando Martínez-Villarino,[b] Luz Diego,[c] Jorge Barroso,[d] Gabriel Merino,*[b] and Zhong-hua Cui.*[a,e]

[a]*Institute of Atomic and Molecular Physics, Jilin University, Changchun 130023, China*

E-mail: zcui@jlu.edu.cn

[b]*Departamento de Física Aplicada, Centro de Investigación y de Estudios Avanzados Unidad Mérida, Km 6 Antigua Carretera a Progreso. Apdo. Postal 73, Cordemex, 97310 Mérida, México.*

[c]*Doctorado en Fisicoquímica Molecular, Facultad de Ciencias Exactas, Universidad Andres Bello, Av. República 275, Santiago, Chile.*

[d]*Department of Chemistry, Clemson University, Clemson, South Carolina, 29634 USA*

E-mail: gmerino@cinvestav.mx

[e]*State Key Laboratory of Inorganic Synthesis and Preparative Chemistry, Jilin University, Changchun 130023, China*

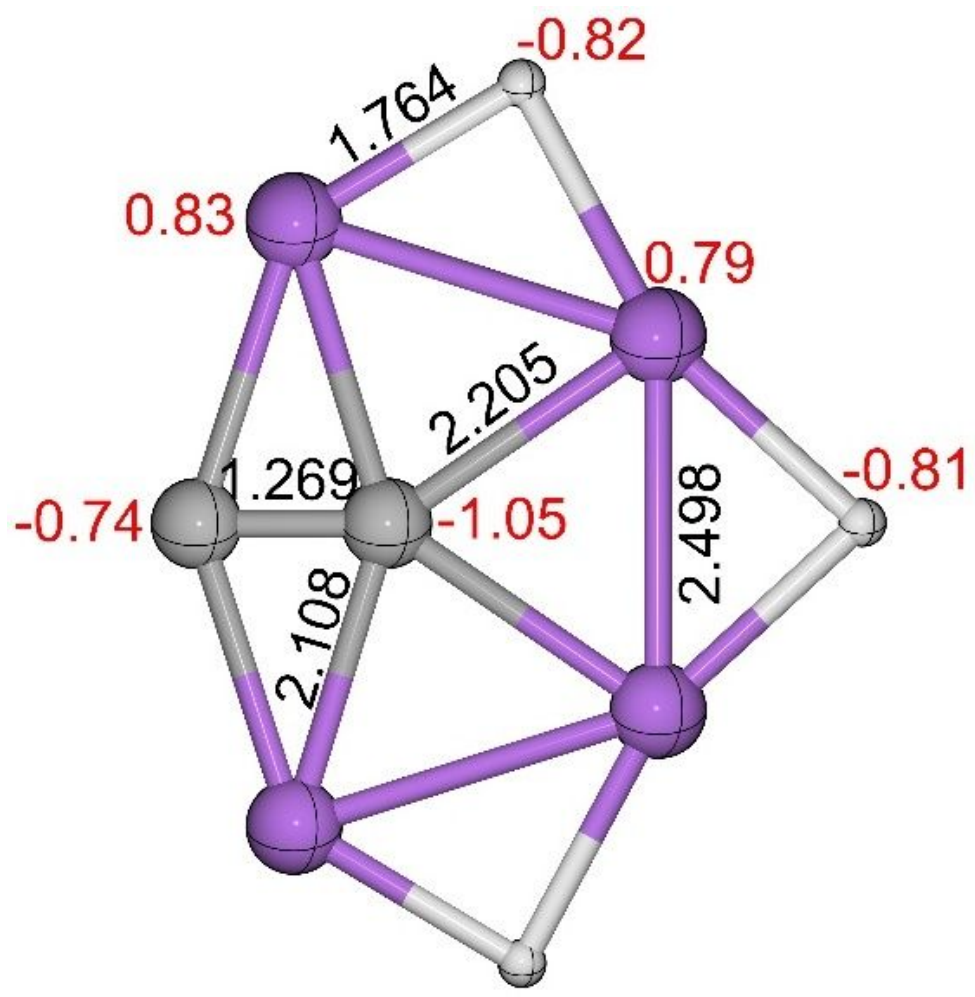

Figure S1. CCSD(T)/aug-cc-pVTZ geometry of $C_2Li_4H_3^-$. Selected bond lengths are given in Å, and NPA charges in |e| are shown in red.

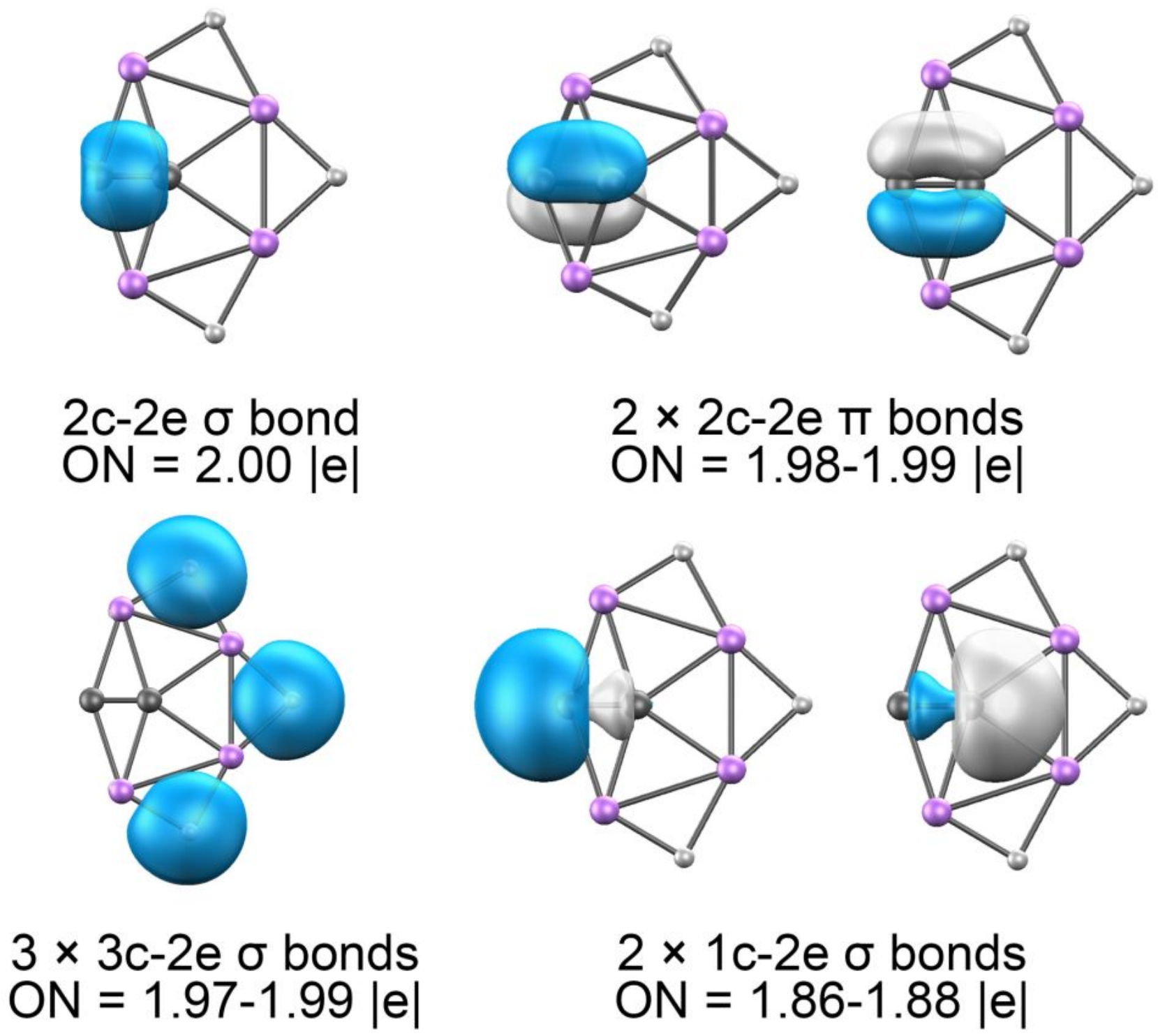

Figure S2. AdNDP analysis of $C_2Li_4H_3^-$ at the PBE0-D3/aug-cc-pVTZ level. Occupation numbers (ONs) are given in |e|.

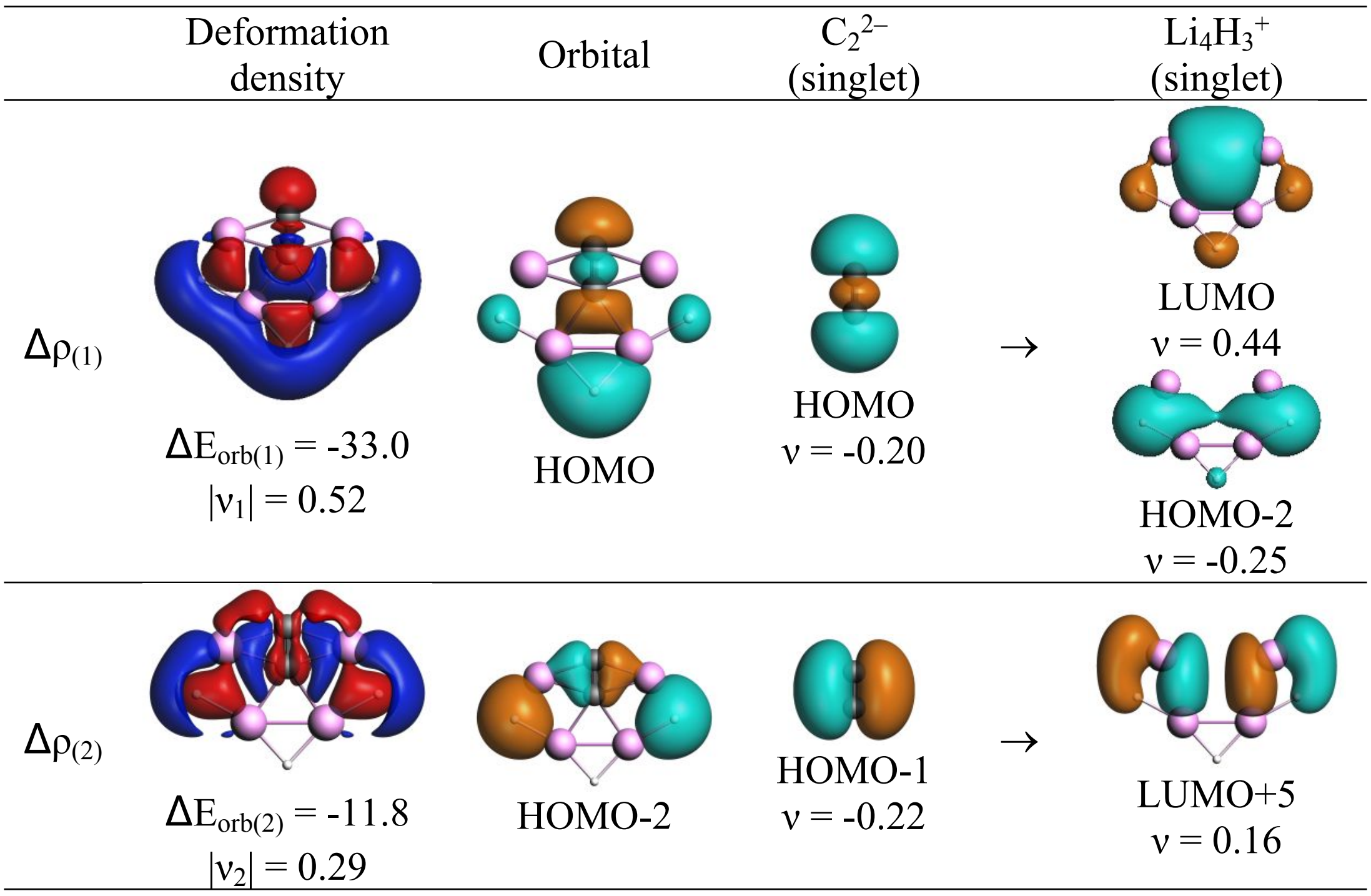

Figure S3. Deformation densities (Δρ) plots of the pairwise orbital interactions in the $C_2Li_4H_3^-$ cluster, along with their interaction energies (ΔE_{orb}, in kcal/mol) and eigenvalues (ν). The eigenvalues indicate the magnitude of charge flow, with the direction represented from red(donor) to blue (acceptor).

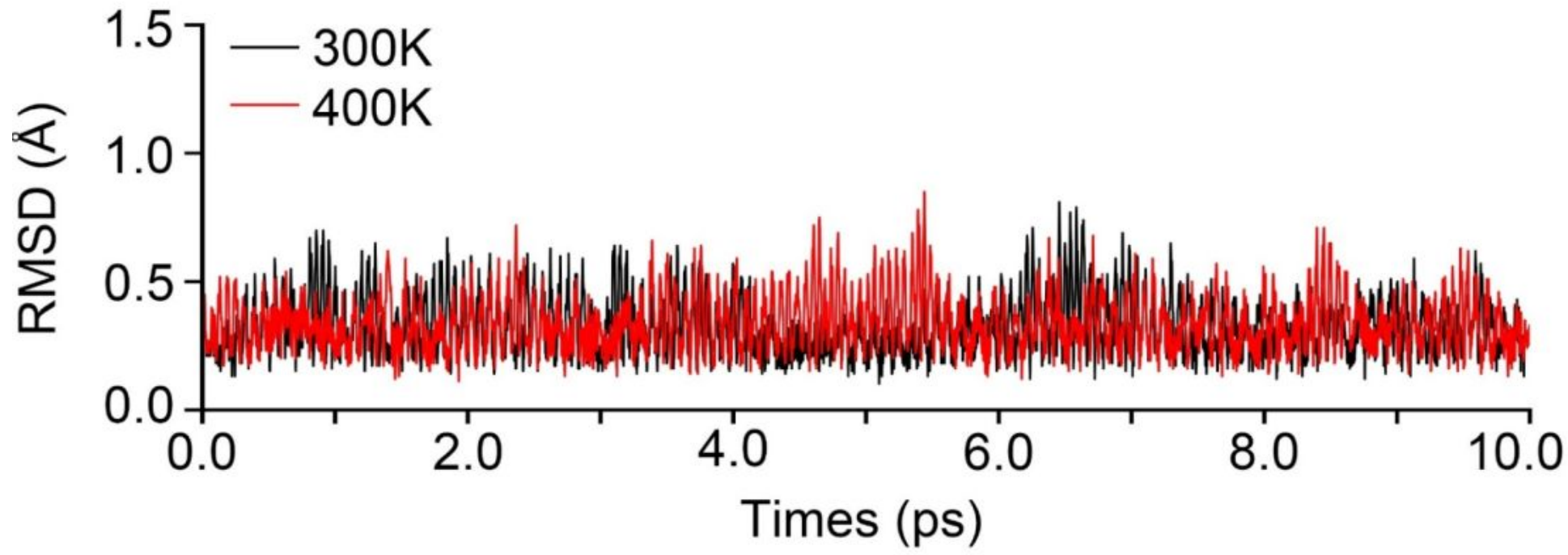

Figure S4. Root-mean-square deviations (RMSD, Å) of $C_2Li_4H_3^-$ obtained from Born-Oppenheimer molecular dynamics simulations at 300 and 400 K over 10 ps trajectories.

Table S1. Energy decomposition analysis (EDA) results for the $C_2Li_4H_3^-$ cluster considering C_2 and Li_4H_3 fragments in different charge and electronic states at the PBE0-D3/TZ2P-ZORA level. Energies are reported in kcal/mol.

Contribution	C_2^{2-} (singlet) $Li_4H_3^+$ (singlet)	C_2^- (doublet) Li_4H_3 (doublet)	C_2(singlet) $Li_4H_3^-$ (singlet)
ΔE_{int}	-408.0	-154.2	-232.9
ΔE_{Pauli}	123.7	227.1	262.2
ΔE_{elstat}	-450.8	-239.3	-223.8
ΔE_{disp}	-0.6	-0.6	-0.6
ΔE_{oi}	-80.4	-141.4	-270.8

Cartesian coordinates of the low-lying energy isomers of $C_2Li_4H_3^-$ optimized at the PBE0-D3/aug-cc-pVTZ level

Frequencies = 42 cm^{-1}
E = -107.9748817 a.u.

Li	0.000000000	1.226233000	-1.619904000
Li	0.000000000	-1.226233000	-1.619904000
Li	0.000000000	1.985484000	0.773786000
Li	0.000000000	-1.985484000	0.773786000
C	0.000000000	0.000000000	1.417728000
C	0.000000000	0.000000000	0.163310000
H	0.000000000	-2.883575000	-0.725195000
H	0.000000000	2.883575000	-0.725195000
H	0.000000000	0.000000000	-2.959127000

Frequencies = 78 cm^{-1}
E = -107.9662161 a.u.

Li	2.078990000	-0.053610000	0.000000000
Li	-2.354893000	-1.316714000	0.000000000
Li	0.077628000	-1.687317000	0.000000000
Li	1.360022000	2.160651000	0.000000000
C	-1.141606000	0.220487000	0.000000000
C	0.000000000	0.725264000	0.000000000
H	3.001572000	1.638530000	0.000000000
H	-1.519627000	-2.814562000	0.000000000
H	1.882450000	-1.807504000	0.000000000

Frequencies = 147 cm^{-1}
E = -107.9644291 a.u.

Li	0.298147000	-2.279874000	0.000000000
Li	-0.825611000	-0.184846000	1.375435000
Li	1.935846000	-0.228009000	0.000000000
Li	-0.825611000	-0.184846000	-1.375435000
C	-0.825611000	1.363405000	0.000000000
C	0.382103000	1.029196000	0.000000000
H	-0.614652000	-1.892646000	-1.578468000
H	-0.614652000	-1.892646000	1.578468000
H	2.142037000	-1.937591000	0.000000000

Frequencies = 65 cm^{-1}
E = -107.9615283 a.u.

Li	-2.265469000	0.603676000	0.000000000

Li	2.536172000	-1.533062000	0.000000000
Li	-0.860416000	-1.431124000	0.000000000
Li	-0.757510000	2.387396000	0.000000000
C	0.885555000	-0.329604000	0.000000000
C	0.000000000	0.549338000	0.000000000
H	3.800082000	-2.678777000	0.000000000
H	-2.586628000	-1.174922000	0.000000000
H	-2.485118000	2.454637000	0.000000000

Frequencies = 151 cm^{-1}
E = -107.965101 a.u.

Li	0.321374000	-0.652009000	1.210549000
Li	-1.545150000	-1.833820000	0.000000000
Li	-1.595923000	0.637367000	0.000000000
Li	0.321374000	-0.652009000	-1.210549000
C	0.321374000	1.185458000	0.000000000
C	1.463265000	0.663792000	0.000000000
H	-1.692159000	-0.696533000	-1.383328000
H	0.171464000	-2.201025000	0.000000000
H	-1.692159000	-0.696533000	1.383328000

Frequencies = 10 cm^{-1}
E = -107.9591006 a.u.

Li	0.114559000	-0.773124000	2.236437000
Li	-0.569948000	2.335530000	0.000000000
Li	0.155500000	-1.882031000	0.000000000
Li	0.114559000	-0.773124000	-2.236437000
C	0.114559000	0.350810000	-0.624192000
C	0.114559000	0.350810000	0.624192000
H	0.163879000	-2.440217000	-1.800026000
H	0.163879000	-2.440217000	1.800026000
H	-1.146474000	3.948958000	0.000000000

Frequencies = 74 cm^{-1}
E = -107.9592252 a.u.

Li	-0.100674000	-0.707193000	1.666871000
Li	-1.441520000	2.370894000	0.000000000
Li	-0.100674000	-0.707193000	-1.666871000
Li	0.896075000	-2.166598000	0.000000000
C	0.835839000	-0.006932000	0.000000000
C	-0.100674000	0.822193000	0.000000000
H	0.236234000	-2.418244000	1.728471000
H	-2.643085000	3.575197000	0.000000000
H	0.236234000	-2.418244000	-1.728471000

Frequencies = 84 cm^{-1}
E = -107.9537932 a.u.

Li	1.746496000	-0.546778000	0.000000000
Li	0.010944000	-1.009343000	1.649301000
Li	0.010944000	2.422206000	0.000000000
Li	0.010944000	-1.009343000	-1.649301000
C	-0.396579000	0.399627000	0.000000000
C	-1.136548000	-0.610262000	0.000000000
H	1.738879000	-1.192988000	1.716260000
H	0.383016000	4.079559000	0.000000000
H	1.738879000	-1.192988000	-1.716260000

Frequencies = 62 cm^{-1}
E = -107.9443826 a.u.

Li	0.000000000	0.923502000	0.000000000
Li	3.557232000	1.105174000	0.000000000
Li	-2.393532000	1.262660000	0.000000000
Li	1.336508000	-1.221051000	0.000000000
C	-0.594483000	-1.195483000	0.000000000
C	-1.665235000	-0.549617000	0.000000000
H	1.945824000	0.432156000	0.000000000
H	-1.098575000	2.436844000	0.000000000
H	5.210432000	1.390740000	0.000000000

Frequencies = 61 cm^{-1}
E = -107.9367352 a.u.

Li	1.054581000	1.499721000	-0.572919000
Li	2.697381000	-0.512333000	-0.207822000
Li	0.728006000	-0.164161000	1.302080000
Li	0.058862000	-1.342705000	-0.866062000
C	-0.710735000	0.571956000	-0.155936000
C	-1.706882000	-0.107914000	0.096161000
H	-2.634269000	-0.576335000	0.340851000
H	2.145134000	0.922850000	0.744945000
H	1.378343000	-1.572330000	0.307025000

Frequencies = 108 cm^{-1}
E = -107.9333377 a.u.

Li	-1.827130000	-2.164250000	0.000000000
Li	0.217888000	-0.737552000	1.343784000
Li	0.217888000	-0.737552000	-1.343784000
Li	-1.767424000	0.324076000	0.000000000
C	0.217888000	0.981551000	0.000000000

C	1.446496000	0.865004000	0.000000000
H	-1.512217000	-1.017805000	1.311760000
H	2.514464000	0.902114000	0.000000000
H	-1.512217000	-1.017805000	-1.311760000
